# Lessons from Agriculture for the Sustainable Management of Malaria Vectors

**DOI:** 10.1371/journal.pmed.1001262

**Published:** 2012-07-10

**Authors:** Matthew B. Thomas, H. Charles J. Godfray, Andrew F. Read, Henk van den Berg, Bruce E. Tabashnik, Joop C. van Lenteren, Jeff K. Waage, Willem Takken

**Affiliations:** 1 Center for Infectious Disease Dynamics and Department of Entomology, Pennsylvania State University, University Park, Pennsylvania, United States of America; 2 Ecology Research Group, Department of Zoology, Oxford University, Oxford, United Kingdom; 3 Department of Biology, Pennsylvania State University, University Park, Pennsylvania, United States of America; 4 Laboratory of Entomology, Wageningen University and Research Centre, Wageningen, The Netherlands; 5 Department of Entomology, University of Arizona, Tucson, Arizona, United States of America; 6 London International Development Centre, London, United Kingdom

## Abstract

Willem Takken and colleagues argue for the expansion of insecticide monotherapy in malaria control by taking lessons from agriculture and including more sustainable integrated vector management strategies.

**Summary Points**

- The effectiveness of insecticide-treated bed nets and indoor insecticide sprays to control adult mosquito vectors is being threatened by the spread of insecticide resistance.
- We argue for expanding beyond “insecticide monotherapy” to more sustainable integrated vector management strategies that use optimal suites of control tactics.
- Experience in agriculture suggests that such integrated approaches can provide more effective and durable pest management.
- This shift will require increased investment in research and translational science.
- Failure to act risks a resurgence of malaria and erosion of community support and donor commitment.

## Vector Control and the Emerging Insecticide Resistance Crisis

The 2011 *World Malaria Report* [1] showed welcome progress in the fight against the world's most important vector-borne disease. In the last 10 years, the estimated incidence of malaria has fallen by 17% globally, with malaria-specific mortality rates reduced by 25%. Central to these gains, especially in Africa, has been the massive scale-up of chemical insecticide interventions against malaria mosquito vectors. Current malaria vector control relies almost exclusively on killing adult mosquitoes with chemical insecticides deployed as either insecticide-treated nets (ITNs) or indoor residual sprays (IRS). However, these technologies use a limited arsenal of insecticides originally developed for agriculture, and their efficacy is threatened by the spread of insecticide resistance [1]–[3]. In 2010, 27 countries in sub-Saharan Africa reported mosquitoes resistant to pyrethroids [1]. Such resistance is alarming because pyrethroids are the only class of insecticides approved for use on ITNs and account for two-thirds of the total product (by area) used in IRS for malaria control [4]. Evidence suggests that resistance is beginning to reduce control [5],[6]. Implementation of alternative management strategies is needed to slow and reverse this trend.

## Parallels with Agriculture

In the middle of the last century, the development of cheap and effective synthetic chemical insecticides revolutionized crop protection. Widespread use of broad-spectrum insecticides reduced pest damage substantially in many systems, prompting discussion of pest eradication, similar to some current discussions of eradication ofmalaria. However, rapid evolution of insecticide resistance, pest resurgence due to disruption of biological control, and harmful environmental side effects quickly revealed the limitations of “pesticide monotherapy” [7]–[9].

The search to find new chemical insecticides continued, stimulated by the transient efficacy of products in use and increased restrictions on available insecticides because of their toxicity to people and other non-target organisms. Meanwhile, academic and government researchers explored ways to reduce reliance on insecticides. In crop systems where insecticide use was actually exacerbating pest problems, researchers combined diverse tools such as pest monitoring and forecasting, conservation of natural pest control, habitat manipulation, and resistant host plants, and thereby limited pesticide use to situations where it was necessary [10]. This approach, called integrated pest management (IPM) [10], reduces the risk of insecticide resistance. IPM is knowledge intensive, relying heavily on farmers' understanding and monitoring of local conditions. Its development therefore engendered a culture of farmer participation and decision-making, providing a balance to the former top-down, technology-driven approach. While not a panacea, IPM is now a cornerstone of many production systems in both developed and developing countries [10]–[14]. Even new technologies, such as genetically engineered crops, can be more effective and sustainable when used with other tactics in IPM [12],[15].

**Figure 1:**
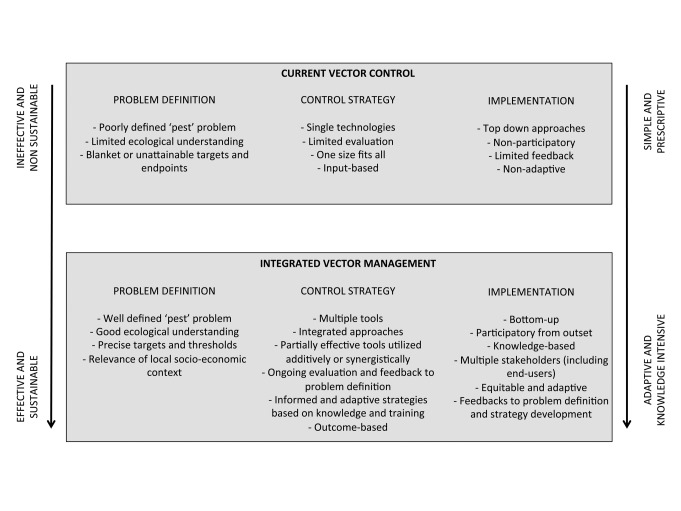
Features of current vector control strategies compared with potential integrated vector management (IVM). The arrows indicate trends representative of the contrasting strategies. Progression towards IVM has the potential to increase the effectiveness and sustainabilty of control, but requires more diverse and knowledge-intensive approaches.

Current malaria vector control has more in common with the agricultural practices of the 1950s than contemporary IPM (Figure 1). There is a reliance locally on single technologies associated with fast-acting insecticides used in ways that impose intense selection pressure for resistance.

The pending resistance crisis creates an urgent need to develop and implement integrated, multi-tactic strategies for vector control that parallel IPM in agriculture. We call this “integrated vector management” (IVM), which we define as the optimal use of diverse tools, tactics, and resources to reduce transmission of disease by vectors. The potential of IVM has been discussed previously (e.g., [16],[17]), and tacit recognition of the approach already exists in World Health Organization (WHO) policy [18],[19]. The transition to more sustainable IVM will, however, require increased efforts in several key areas.

## Quantifying the Problem

One of the foundations of IPM and thus IVM is to quantify the “pest” or “vector” problem and define the targets for control. For malaria this might seem straightforward—“control mosquitoes and reduce disease as much as possible”. Yet, it is surprising how little is understood about how local vector ecology contributes to infection. A typical list of unknowns could include the temporal and spatial distribution of biting, rate of parasite development, local variation in vector competence, sites where mosquitoes rest, the causes and rate of adult mosquito mortality, the nature of density-dependent regulation, and sometimes even which vector species is most important [20],[21]. Equally little is understood regarding the impact of insecticide resistance on vectorial capacity and malaria epidemiology [22],[23]. These unknown factors influence the approaches and strategies required to reduce malaria transmission in a particular setting. For example, while a 30% reduction in infectious bites might substantially reduce disease prevalence in a low transmission environment, even a 90% reduction might not be sufficient in a high transmission environment [24]. Effective IVM requires a better understanding of local vector and transmission ecology with appropriate targets for control defined in ways analogous to economic thresholds of pest density used widely to guide pest control decisions in agriculture.

## Conventional Chemicals

Highly lethal insecticides like pyrethroids knock down and kill mosquitoes rapidly after contact. This lethality can provide excellent disease control, yet it also selects intensely for resistance. Development of replacement insecticides is one recognized strategy to address this problem [25]. However, the insecticide target product profiles prescribed by the WHO Pesticide Evaluation Scheme (WHOPES) set a high bar with respect to rapid killing, high persistence, and low mammalian toxicity. This, together with protracted regulatory procedures, means new insecticides are still many years off [3]. Moreover, novel chemistry will not prevent resistance evolution [26]. Resistance management strategies used in agriculture such as insecticide combinations and rotations require two or more insecticides with diverse modes of action to avoid cross-resistance [27], yet this diversity is not commonly available for vector control [28]. This problem is compounded when the same insecticide active ingredients are used in both agriculture and vector control [29],[30]. In the only controlled trial of resistance management strategies for malaria mosquito vectors we know of, rotations or mosaics did not delay pyrethroid resistance [26],[31].

In addition, ITNs and IRS only target mosquitoes inside domestic dwellings, leaving potentially significant fractions of the vector community untouched. While outdoor biting tends to be less epidemiologically important than indoor biting, it still contributes to transmission [32],[33]. Thus, even in the absence of resistance, it is unlikely that ITNs and IRS will be sufficiently effective to meet the goal of long-term malaria suppression in intense transmission settings.

## Additional Tools

Current vector control relies on killing mosquitoes quickly with neurotoxins. However, more subtle approaches, such as slow-acting insecticides that shorten adult mosquito longevity, could also reduce transmission while imposing less intense selection for resistance [24],[34]. Alternative modes of action that impair olfaction, flight, energy metabolism, or immunity could further contribute to reduced vectorial capacity (e.g., see [35]). Such “sub-lethal insecticides” would represent genuinely new additions to the mosquito control tool kit that extend beyond the current fast-acting insecticide paradigm [36].

In addition, chemical insecticides that act against the adult vectors are not the only available tools. Physical barriers such as house screens [37], habitat management to reduce vector breeding site quality [38], microbial larvicides [39], and manipulation of nectar sources [40] could contribute to reduced disease transmission. Other tools in development such as fungal biopesticides [41], odor-baited traps [42], manipulation or release of parasites [43], and genetically modified [44],[45] or transinfected mosquitoes [46] could add to the list.

Individually, many of these technologies face today the same constraints that alternatives to insecticides faced in crop protection: marketing and regulatory systems for new products favored broad spectrum, fast-acting, lethal insecticides that provided stand alone, albeit unsustainable, solutions to pest problems. Against this model, subtler alternative methods cannot compete, except in an IPM/IVM context, where the benefit comes from the sum of the parts. It is important that regulatory frameworks are amenable to IVM to encourage research and development (R&D) and prevent barriers to ultimate commercialization.

## Integrated Strategies and Sustainable Implementation

Developing effective IVM will require better understanding of the impact of control tactics individually and in various combinations [39],[47]–[49]. Again, there is surprisingly little relevant research. Yet, different combinations of tools could deliver the same end points with strategies optimized over time and space.

Development of IVM will also require substantial money and effort. It has been estimated that effective delivery of ITN or IRS measures will require 40%–61% of projected national malaria control program budgets [50]. This is in sharp contrast to the 4% of the global malaria R&D budget that is currently spent on vector control [51]. Given the historic and contemporary significance of vector control in reducing malaria [52], this level of funding is inadequate. Experience from agriculture suggests that with appropriate engagement and education, even complex knowledge-intensive practices can be successfully implemented. Extensive IPM programs in many developing countries indicate that such strategies are best developed and implemented via bottom-up approaches engaging end users from the outset in research and development [53],[54]. Embracing this philosophy can bolster vector control and move it away from top-down prescriptions towards adaptive, surveillance-, and evidence-based strategies that vary in space and time depending on local conditions. As with IPM, IVM can be best advanced by engaging the end users and working in partnerships to generate shared knowledge and solutions relevant to the local context. This strategy is necessary not only to develop effective solutions, but also to avert the risks of donor and community fatigue. There is no “quick fix” for sustainable vector control, or for eradication of malaria.

## Conclusions

Ensuring continued advance in malaria control requires rethinking how we manage vector populations. Current strategies rely heavily on repeated application of single neurotoxic insecticides that quickly kill adult mosquitoes. This narrow paradigm is beginning to fail, as it did in agriculture, as well as in previous malaria eradication campaigns of the '50s and '60s. We should not abandon ITNs and IRS; these can be useful in IVM just as insecticides are in IPM. But experience with IPM in agriculture suggests that integrated approaches have the potential to provide more *effective* and *durable* pest management. To achieve the equivalent for malaria control requires additional tools in the armory, a better understanding of the impact of individual tools and their interactions, appropriate training for end users, and design of novel integrated strategies that maximize impact and fit the local ecological and socioeconomic context. Given the current lack of any clear alternative to the current insecticide paradigm, researchers, policy makers, and funding agencies need to act now to support this more diverse and adaptive ap proach. It is unlikely that any single tactic or combination of tactics will provide a permanent solution. Vector control programs must proactively and continuously innovate to optimize and sustain impact.

## Abbreviations

IPM: integrated pest management
IRS: indoor residual sprays
ITN: insecticide-treated net
IVM: integrated vector management
R&D: research and development
WHO: World Health Organization
